# Treatment of Cerebro-Spinal Meningitis

**Published:** 1883-06-20

**Authors:** 


**Treatment of Cerebro-Spinal Meningitis.**—Prof. H. C. Wood, in a clinical lecture in the Medical Gazette, sums up as follows: During the first three or four days in the strong and robust, leeches or cups may be applied to the temples or nape and upper part of the spine. Ice-bags are applied to the head and back of neck for the first days—in many for a week. To relieve headache, restlessness and delirum bromide of potash is the best agent, gr. 20 to 30 every three hours. Its efficacy is increased by adding chloral (ten grain doses usually) or in those who cannot take chloral, tinct. hyosyami (drachm doses) in the hysterically inclined. If possible don't use opium, but sometimes it becomes necessary, as the remedies already named occasionally fail. The temperature is not apt to run over 104° (a very harmless height) in adults except at the close. and quinine is not indicated; moreover, it has no effect in lowering the temperature; if this be an object, it is best done bycold affusions, cold and tepid baths, or the cold pack.—*Ex.*

